# Decreases in Colonic and Systemic Inflammation in Chronic HIV Infection after IL-7 Administration

**DOI:** 10.1371/journal.ppat.1003890

**Published:** 2014-01-30

**Authors:** Irini Sereti, Jacob D. Estes, William L. Thompson, David R. Morcock, Margaret A. Fischl, Thérèse Croughs, Stéphanie Beq, Sylvie Lafaye de Micheaux, Michael D. Yao, Alexander Ober, Eleanor M. P. Wilson, Ven Natarajan, Hiromi Imamichi, Mohamed R. Boulassel, Michael M. Lederman, Jean-Pierre Routy

**Affiliations:** 1 National Institute of Allergy and Infectious Diseases, National Institutes of Health, Bethesda, Maryland, United States of America; 2 Frederick National Laboratory for Cancer Research, Leidos Biomedical Research, Inc, Frederick, Maryland, United States of America; 3 University of Miami School of Medicine, Miami, Florida, United States of America; 4 Cytheris, Paris, France; 5 McGill University Health Center, Montreal, Quebec, Canada; 6 Case Western Reserve University, Cleveland, Ohio, United States of America; Emory University, United States of America

## Abstract

Despite antiretroviral therapy (ART), some HIV-infected persons maintain lower than normal CD4^+^ T-cell counts in peripheral blood and in the gut mucosa. This incomplete immune restoration is associated with higher levels of immune activation manifested by high systemic levels of biomarkers, including sCD14 and D-dimer, that are independent predictors of morbidity and mortality in HIV infection. In this 12-week, single-arm, open-label study, we tested the efficacy of IL-7 adjunctive therapy on T-cell reconstitution in peripheral blood and gut mucosa in 23 ART suppressed HIV-infected patients with incomplete CD4^+^ T-cell recovery, using one cycle (consisting of three subcutaneous injections) of recombinant human IL-7 (r-hIL-7) at 20 µg/kg. IL-7 administration led to increases of both CD4^+^ and CD8^+^ T-cells in peripheral blood, and importantly an expansion of T-cells expressing the gut homing integrin α4β7. Participants who underwent rectosigmoid biopsies at study baseline and after treatment had T-cell increases in the gut mucosa measured by both flow cytometry and immunohistochemistry. IL-7 therapy also resulted in apparent improvement in gut barrier integrity as measured by decreased neutrophil infiltration in the rectosigmoid lamina propria 12 weeks after IL-7 administration. This was also accompanied by decreased TNF and increased FOXP3 expression in the lamina propria. Plasma levels of sCD14 and D-dimer, indicative of systemic inflammation, decreased after r-hIL-7. Increases of colonic mucosal T-cells correlated strongly with the decreased systemic levels of sCD14, the LPS coreceptor - a marker of monocyte activation. Furthermore, the proportion of inflammatory monocytes expressing CCR2 was decreased, as was the basal IL-1β production of peripheral blood monocytes. These data suggest that administration of r-hIL-7 improves the gut mucosal abnormalities of chronic HIV infection and attenuates the systemic inflammatory and coagulation abnormalities that have been linked to it.

## Author Summary

HIV infected people who receive antiretroviral therapy (ART) remain at higher risk of non-infectious complications such as cardiovascular disease. This risk is linked to persistent inflammation and immune activation and is higher in those with lower circulating CD4^+^ T-cell counts. IL-7 therapy can increase CD4^+^ and CD8^+^ T-cell counts in peripheral blood, but its effects on gut mucosal T cell restoration was unknown. We gave 3 doses of interleukin (IL)-7 to HIV^+^ persons receiving ART and performed gut biopsies before and after therapy. IL-7 therapy increased T-cell numbers in the periphery including T-cells that express the α4β7 integrin, a molecule that promotes trafficking to the gut. Gut mucosa studies showed significant increases of T-cells and significant decreases in local inflammation as reflected by expression of myeloperoxidase and tumor necrosis factor. In addition, plasma levels of inflammatory markers that are linked to mortality in HIV infection decreased significantly as did some inflammatory monocyte markers. We conclude that IL-7 administration can improve gut mucosal abnormalities in chronic treated HIV infection and as a result, also may decrease both gut and systemic inflammation.

## Introduction

Mucosal CD4^+^ T-cell depletion in the gut is a hallmark of HIV pathogenesis [1]. CD4^+^ T-cells residing in the gut mucosa are predominantly of memory phenotype and are frequently activated due to constant antigenic exposure. They typically express CCR5, the major HIV co-receptor, and α4β7, an integrin that assists gut homing of T-cells and can also facilitate HIV transmission [2], [3]. The CD4^+^ T-cell depletion in the gut has been implicated in the pathogenesis and persistence of immune activation in untreated and chronically treated HIV infection [4], [5]. The loss of epithelial integrity that accompanies the CD4^+^ T-cell loss leads to continuous activation of the innate system. Evidence of increased microbial translocation as reflected by increased systemic levels of bacterial lipopolysaccharide (LPS) or 16S DNA [4], [6] has not been directly linked to clinical events in HIV infected patients treated with antiretroviral therapy (ART). On the other hand, biomarkers associated with monocyte activation (sCD14) as well as inflammation (IL-6) and coagulation (D-dimer) have been consistently found to be independent predictors of morbidity and mortality in this patient population [7]–[9]. Administration of ART can repair to a significant degree the HIV-inflicted gut mucosal injury [10], [11] but studies suggest that CD4^+^ T-cells in the lamina propria (LP) may not be fully restored to the levels observed in uninfected controls. Recent evidence also suggests that defective T-cell trafficking, regulated by chemokines and their respective receptors, may be responsible for the altered T-cell homeostasis in the gut mucosa of HIV-infected persons after ART [12], [13].

Prolonged ART has dramatically improved the survival of HIV infected patients and has shifted the morbidity of the epidemic from infectious opportunistic diseases to non-infectious events that include cardiovascular, hepatic and renal disease and non-AIDS malignancies [14], [15]. Immunologic non-responders (patients who maintain a lower peripheral CD4^+^ T-cell counts despite sustained suppression of plasma HIV viremia) remain at higher risk for both AIDS and non-AIDS clinical events [16]. Immune-based therapies are thus still pursued in chronic HIV disease with the goal of improving both CD4^+^ T-cell restoration and overall immune competence of HIV-infected individuals, thereby reducing chronic immune activation and the associated risk of non-infectious chronic complications in ART-treated patients.

Interleukin 2 (IL-2) had been studied earlier in the past as an ART adjuvant and despite successful CD4^+^ T-cell expansions in peripheral blood, failed to confer any clinical benefit in large adequately powered phase III clinical trials [17]. IL-2 had expanded predominantly CD25^+^ T-cells sharing features of Treg and did not restore gut mucosal CD4^+^ T-cells [18], [19]. In addition during IL-2 administration, there was evidence of transient increased inflammation and activation of the coagulation cascade with elevated levels of IL-6 and D-dimer [20]. Grade IV thrombotic events were reported during the phase III studies in the IL-2 arm suggesting a possible clinical correlate of the biomarker observations [17].

IL-7 has emerged as a candidate immune-based therapy that could succeed in expanding T-cells without a predilection for Treg cell expansion and without significant induction of pro-inflammatory cytokines during administration [21]. Although phase I studies have addressed the safety profile and biologic activity of recombinant human IL-7 (r-hIL-7) by demonstrating T-cell cycling and T-cell expansions [22], [23], the potential impact of r-hIL-7 on mucosal T-cell restoration has not been addressed to date. It was recently demonstrated that IL-7 induces expression of α4β7 in human T-cells both in vitro and in vivo, a phenomenon that can lead to homing of naïve T-cells to the gut in both murine and nonhuman primate models [24], [25].

Based on these observations we designed a clinical trial of r-hIL-7 administration in HIV-infected patients who were receiving ART, maintained a plasma HIV level <50 copies/mL and had inadequate immunologic restoration with circulating CD4^+^ T-cells of 101–400/µL. The objectives of the study were to evaluate the T-cell changes both in the peripheral blood and the gut mucosa and to measure biomarkers that have been associated with clinical events in treated HIV infection. We report here that one cycle of r-hIL-7, consisting of three subcutaneous injections, was well tolerated and led to significant and sustained CD4^+^ and CD8^+^ T-cell expansions in peripheral blood. In addition, gut mucosal T-cells increased and gut mucosal inflammation decreased with decreased neutrophil infiltration and expression of TNF. Systemic levels of biomarkers (sCD14 and D-dimers) that have both been strongly linked to mortality in ART-treated persons also diminished.

## Results

### Participant characteristics at baseline

The participant characteristics at study enrollment are shown in Table 1. Of 36 persons who were screened, 23 enrolled. Screening failures were mainly due to reactivity with HIV-2 antigens on immunoassays (n = 5), HIV-RNA >50 copies/mL (n = 4), >400 CD4^+^ T-cells/µL (n = 3) and abnormal levels of thyroid stimulating hormone (n = 3). The median age at study enrollment was 47 (IQR 41–51) and the majority of participants were white men. The median CD4^+^ T-cell count at baseline was 263 cells/µL and the median ART duration was 5 years. Twelve participants underwent rectosigmoid research biopsies at baseline, prior to any r-hIL-7 administration, and at study week 12 (one was done at week 24). The baseline characteristics of the gut biopsy participants did not differ from those of the overall study population.

**Table 1:** Participant characteristics at baseline (N = 23).

Characteristic	
Age, years	47 (41–51)
Gender – Male/Female	20 (87%)/3 (13%)
Race	
White	16 (69.6%)
Black	3 (13.0%)
Other	4 (17.4%)
Years since HIV diagnosis	9 (7–24)
Years on ART	5 (4–17)
Nadir CD4^+^ T-cells/µL	41 (19–163)
CD4^+^ T-cells/µL	263 (191–329)
CD8^+^ T-cells/µL	604 (350–876)
Plasma HIV-RNA	<50 copies/mL

Median values with IQR or N (%).

### Increased CD4^+^ and CD8^+^ T-cells expressing the integrin α4β7 in peripheral blood

**Figure 1:**
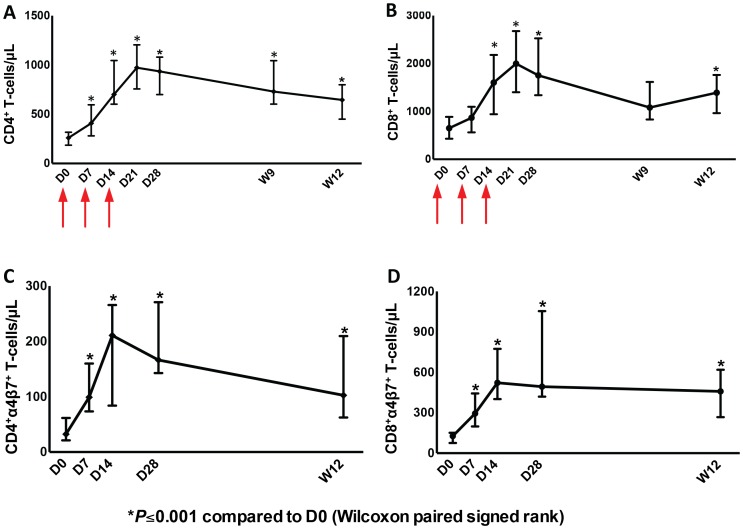
Increases of CD4^+^ and CD8^+^ T-cells as well as CD4^+^ and CD8^+^ T-cells expressing α4β7 in peripheral blood after administration of r-hIL-7. Graphs represent CD4**^+^** (A) and CD8**^+^** (B) T-cells/µL over time after drug administration at days (D) 0, 7 and 14 (arrows), CD4. Significant T-cell increases were observed at day 28 (P<0.001) that persisted to week 12. Day 0 (D0) was the day of the first r-hIL-7 injection. Similarly, CD4**^+^** (C) and CD8**^+^** (D) T-cells/µL expressing α4β7 increased significantly after r-hIL-7 administration. P values represent Wilcoxon paired signed rank tests.

As was seen in earlier studies, administration of one cycle of r-hIL-7 consisting of 3 subcutaneous injections of 20 µg/kg each given once weekly, led to significant and sustained expansion of both CD4^+^ and CD8^+^ T-cells at week 12. Circulating CD4^+^ T-cells increased from 260 to 645 cells/µL and CD8^+^ T-cells increased from 650 to 1390 cells/µL at week 12, both highly significant, P<0.001 in paired comparison (Figure 1A and B). Further phenotypic analysis showed that both naïve and memory peripheral T-cells expanded consistent with previous reports (Figure S1A and S1B). In agreement with the previous study [26], CD4^+^ T-cells with a Treg phenotype (FOXP3^+^ and expressing high levels of CD25) decreased as a proportion of total CD4^+^ T-cells while their absolute number increased (data not shown). As the main objective of this study was to evaluate effects of r-hIL-7 on the colonic mucosa, expression of α4β7was also evaluated in peripheral blood T-cells. The proportion of CD4^+^ T-cells expressing α4β7 increased from 15.4% at baseline to 26.2% at day 14 and 17.7% at week 12 (both at P<0.001). The proportion of CD8^+^ T-cells expressing α4β7 also increased from 17.3% at baseline to 34.3% at day 14 and to 29.6% at week 12 (both at P<0.001). This led to an overall increase in the number of CD4^+^ T-cells expressing α4β7 from 32 cells/µL at baseline to 211 cells/µL at day 14 and to 103 cells/µL at week 12 (Figure 1C, P = 0.001). The CD8^+^ T-cells expressing α4β7 increased from 127 cells/µL at baseline to 522 cells/µL at day 14 and to 458 cells/µL at week 12 (Figure 1D, P = 0.001). Increases were seen in naïve, central and effector memory T-cells but were more pronounced in naïve T cells (Figure S2A and S2B).

### Increased T-cells in the gut mucosa after r-hIL-7 administration

**Figure 2:**
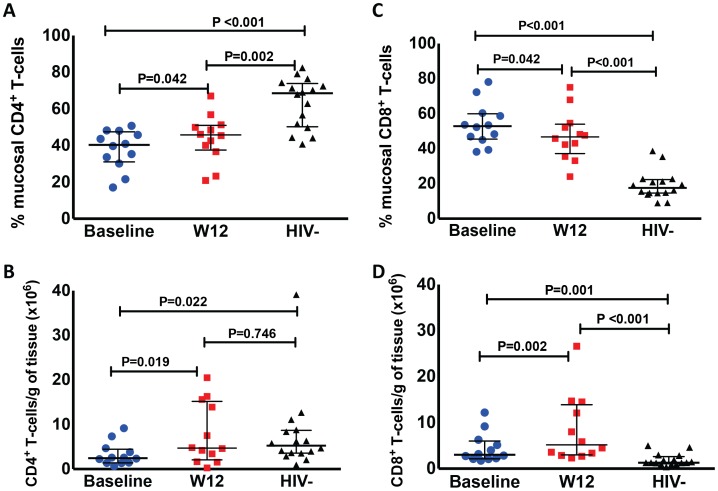
Administration of r-hIL-7 leads to increases in gut mucosal T-cells. The proportion of CD4**^+^** (A) and CD8**^+^** (C) T-cells in gut tissue was assessed after gating in CD3+ cells at baseline (prior to r-hIL-7) and at week 12 showing an increase in the proportion of CD4**^+^** T cells (P = 0.042) and a decrease in the proportion of CD8**^+^** T-cells (P = 0.042). Comparisons of HIV+ participants with HIV^−^ controls were done by Mann-Whitney testing. The number of CD4**^+^** (B) and CD8**^+^** (D) T-cells per gram of gut tissue was also calculated as described in methods and showed increases in the numbers of CD4^+^ and CD8^+^ T-cells (P = 0.019 and P = 0.002 respectively) as well as normalization of CD4^+^ T-cell numbers compared to HIV^−^ controls' numbers at week 12 (P = 0.022 at baseline and P = 0.746 at week 12 by Mann-Whitney test). Baseline indicates prior to any drug administration, gut biopsies were performed between 1 and 3 weeks prior to the first r-hIL-7 injection at day 0 (D0).

The proportion of CD4^+^ T-cells (gated in CD3^+^ cells) in the colonic mucosa was lower in HIV^+^ participants compared to HIV− controls (P<0.001 by Mann-Whitney test). The CD4^+^ T-cell proportion increased in nearly all (11 out of 12) study participants who underwent biopsies at week 12 from a median of 40.3% to 45.4% after r-hIL-7 (P = 0.042 by paired signed rank test, Figure 2A) but remained significantly lower compared to controls (P = 0.002 by Mann-Whitney test). This increase was also observed when absolute numbers of CD4^+^ T-cells per gram (g) of rectosigmoid tissue were measured as previously described [11] from 2.5 to 4.8×10^6^ cells/g tissue (P = 0.019, Figure 2B) leading to normalization when compared to numbers among HIV^−^ controls (P = 0.022 before r-hIL-7 and P = 0.746 at week 12 by Mann-Whitney test, Figure 2B).

**Figure 3:**
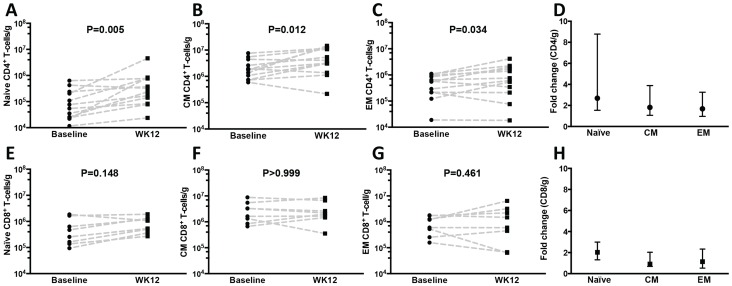
Naïve, central and effector memory colonic mucosal CD4^+^ T cells increase after r-hIL-7 administration. The phenotype of gut mucosal T-cells was further evaluated by CD45RO and CD27 staining and revealed significant increases at week 12 of (A) naïve (CD45RO^−^/CD27^+^), (B) central (CD45RO^+^/CD27^+^), and (C) effector (CD45RO^+^/CD27^−^) memory CD4**^+^** T-cell numbers compared to baseline (Wilcoxon paired signed rank test) with overall higher fold-increases of naïve cells (D). The differences in CD8^+^ T-cell subsets (N = 8) did not reach statistical significance (E–H). Baseline indicates prior to any drug administration, rectosigmoid biopsies were performed between 1 and 3 weeks prior to the D0 r-hIL-7 injection.

The proportion of CD8^+^ T-cells (gated in CD3^+^ cells) in the colonic mucosa was 52.9% at baseline (significantly higher than among HIV− controls, P<0.001), and decreased to 45.8% at week 12 (P = 0.042 by paired signed rank test, Figure 2C) but remained higher than among controls (P<0.001). The numbers of gut CD8^+^ T-cells increased overall from 3×10^6^/g to 5.9×10^6^/g of tissue (P = 0.002 by paired signed rank test, Figure 2D). The CD8^+^ T-cell numbers were higher in HIV^+^ patients compared to controls both at baseline and at week 12 (P = 0.001 and P<0.001 respectively by Mann-Whitney test, Figure 2D). The proportion of gut γδ T-cells decreased (6.1 to 3.4%, P = 0.005) but their number/g of tissue did not change significantly (P = 0.365). The phenotype of mucosal T-cells at week 12 consisted of naïve, central memory and effector memory cells and the numeric increases reached statistical significance for all of the CD4^+^ (N = 12) but not for the CD8^+^ T-cell subsets (N = 8) (Figure 3A–F). CD4^+^ T-cell changes in colonic mucosa from baseline to week 12 correlated with changes in circulating CD4^+^α4β7^+^ T-cells (r = 0.65, P = 0.026). In contrast, an association between changes in colonic CD8^+^ T-cells with peripheral blood CD8^+^α4β7^+^ T-cells was not observed (P = 0.991) (Figure S2C and S2D).

The proportion of cycling (Ki67^+^) gut mucosal CD4^+^ and CD8^+^ T-cells did not change at week 12 (P = 0.831 and P = 0.193 respectively). When assessed as absolute numbers, both CD4^+^Ki67^+^ (P = 0.024) and CD8^+^Ki67^+^ T-cells (P = 0.037) increased from baseline to week 12 (Figure S3A and S3B). In addition, the number of CD8^+^, but not CD4^+^, Ki67^+^ T-cells/g of tissue at week 12 inversely correlated with the proportion of mucosal cycling CD8^+^ T-cells (r = −0.90, P<0.001 by Spearman rank, Figures S3C and S3D). Cytokine production after mitogenic stimulation showed an overall decrease in the proportion of IFNγ^+^ (from 22.36 to 12.99%, P = 0.002), TNF^+^ (from 42.25 to 39.06%, P = 0.520) and IL-17^+^ (from 7.00 to 4.73%, P = 0.077) CD4^+^ T-cells (Figure S4A–C). An overall, non-statistically significant, decrease in the proportion of FOXP3^+^ CD4^+^ T-cells in rectosigmoid mucosa was also noted (from 5.86 to 4.57%, P = 0.065, Figure S4D). When FOXP3 analysis was performed in mucosal CD3^+^ cells, the percent did not change after r-hIL-7 (4.2% at baseline vs 4.8% at week 12, P = 0.375) but the number of CD3^+^FOXP3^+^ cells/g increased from 39.0×10^4^ cells/g at baseline to 90.1×10^4^ cells/g at week 12 (P = 0.001).

### Increased T-cells and decreased neutrophils (MPO^+^) in LP by immunohistochemistry after r-hIL-7 administration

**Figure 4:**
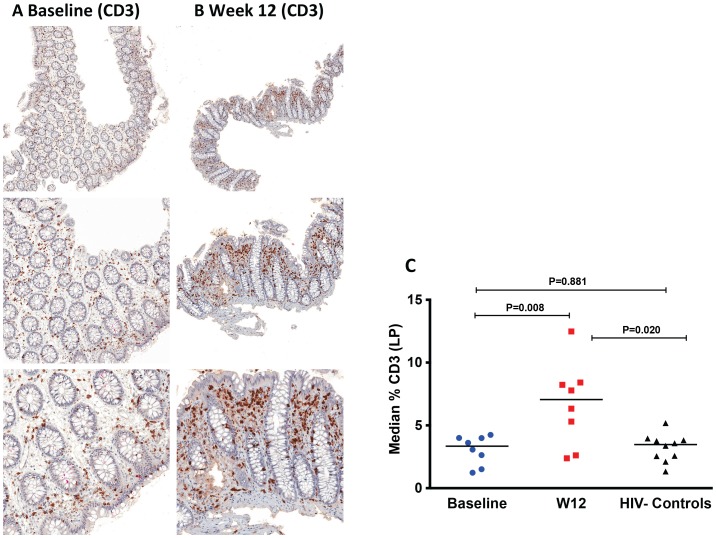
Increase of CD3^+^ cells in the lamina propria (LP) by immunohistochemistry at week 12 after r-hIL-7 administration. The percent area of the LP staining for CD3 was evaluated at both baseline, pre-IL-7 (A), and at week 12, post-IL-7, (B) as shown in representative IHC images from one study participant with brown stained cells depicting CD3**^+^** cells at 50× (top panel), 100× (middle panel) and 200× (bottom panel) images. (C) Study participants had a significant increase in CD3**^+^** cells at week 12 compared to baseline (P = 0.008 by paired signed rank test) leading to increased CD3^+^ surface area compared to surface areas of controls (P = 0.020, Mann-Whitney test). Baseline indicates prior to any drug administration, gut biopsies were performed between 1 and 3 weeks prior to the first (D0) r-hIL-7 injection.

The changes in colonic mucosal T-cells after r-hIL-7 were further evaluated by immunohistochemistry in a subset (N = 8) of study participants. The surface area stained for total T-cells (CD3) and each T cell type using specific phenotypic markers (CD4 and CD8) was compared in study participants between baseline and week 12, after r-hIL-7 administration. It has been shown previously that the percent area staining for CD4 (and other cellular markers) is directly proportional to the number of cells per unit area of tissue [27]. We applied this method to understand T-cell density changes as well as the level of neutrophil infiltration within the lamina propria (LP) after r-hIL-7 treatment. As demonstrated in Figure 4, the percent area of LP staining for CD3^+^ cells was similar in HIV^+^ participants and HIV^−^ controls at baseline (P = 0.881) but increased significantly after r-hIL-7 (P = 0.008 by paired signed rank test) and became higher than among controls at week 12 (P = 0.020 by Mann-Whitney test). The change in CD3^+^ cells measured by IHC correlated with the change in CD3^+^ cells measured by flow cytometry (r = 0.952, P = 0.001). Staining for CD4^+^ and CD8^+^ cells was subsequently done separately in LP sections and showed statistically significant increases in CD8^+^ but not CD4^+^ T cells (P = 0.008, Figure S5, A and B). Enumeration of T cells in lymphoid follicles was not feasible by IHC due to rare numbers of follicles in the biopsied tissue and the limited number of tissue samples processed for IHC.

**Figure 5:**
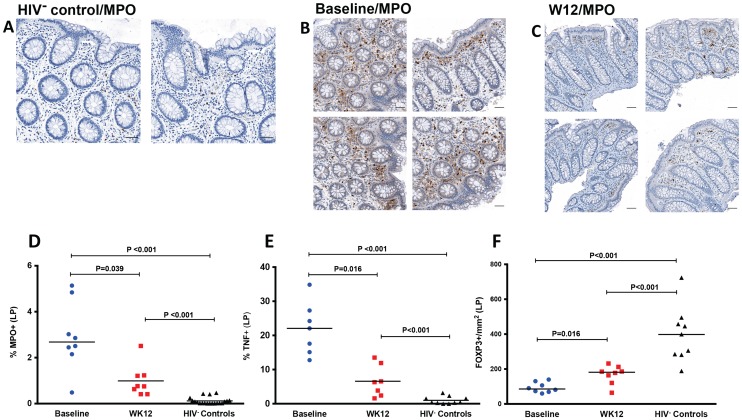
Decreased neutrophil infiltration by myeloperoxidase (MPO) staining, decreased TNF and increased FOXP3 in LP at week 12 after r-hIL-7 administration. Examples of IHC images of MPO staining in an HIV− control (A), a study participant prior to r-hIL-7 administration (B) and at week 12 after r-hIL-7 (C). Summary results of the percent area of the LP staining for MPO (D) showing a significantly higher number of neutrophils in HIV^+^ samples than in HIV^−^ controls' (P<0.001) and a decrease at week 12 after r-hIL-7 administration compared to baseline (P = 0.039) that remained different from HIV^−^ controls (P<0.001). The percent area of LP staining for TNF (E) was also higher in HIV^+^ participants at baseline than among HIV^−^ controls (P<0.001), and decreased significantly after r-hIL-7 (P = 0.016 by Wilcoxon matched pairs rank). In contrast, intracellular FOXP3 staining/mm^2^ in LP (F) was significantly lower in HIV^+^ participants than among controls (P<0.001) and increased significantly at week 12 (P = 0.016). Baseline indicates prior to any drug administration, gut biopsies were performed between 1 and 3 weeks prior to the first r-hIL-7 injection.

Immunohistochemical staining for myeloperoxidase (MPO) was also performed to get an estimate of myeloid (neutrophil) cell infiltration, which is considered indicative of gut epithelial barrier damage and microbial translocation [28]. The proportion of the LP area staining MPO^+^ was significantly higher in samples of HIV^+^ participants at baseline than in biopsies obtained from HIV^−^ controls (P<0.001, Figure 5A, 5B and 5D), consistent with sustained gut damage and microbial translocation in these ART suppressed patients. At week 12 after r-hIL-7 administration, a significant decrease of MPO staining was noted in the LP of the HIV^+^ study participants (P = 0.039, Figure 5 C–D and Figure S6A), but levels remained higher than those measured in the LP of HIV^−^ controls (P<0.001, Figure 5D).

In order to further characterize the mechanisms of decreased local inflammation, TNF and FoxP3 staining was also done by IHC. The percent area of the LP staining for TNF, as shown in Figure 5E, was higher in HIV^+^ patients (N = 7) than in HIV^−^ controls (P<0.001) and decreased significantly after r-hIL-7 administration (P = 0.016 by paired rank test, Figure S6B), remaining higher than among controls (P<0.001). FOXP3 staining in LP was significantly lower at baseline in the HIV^+^ subjects than among HIV^−^ controls (P<0.001) and increased significantly (P = 0.016) after r-hIL-7 administration but did not achieve levels seen among controls (Figure 5F and Figure S6C). A strong correlation was observed between the change in TNF staining after r-hIL-7 and the changes in FOXP3 staining (r = 0.89, P = 0.033 by Spearman rank) (Figure S6D) but not between MPO and TNF or FOXP3.

In a small subset of HIV^+^ participants (N = 3), cryopreserved gut tissue was available and used for exploratory RNA extraction and measurements of mRNA levels for selected chemokines, chemokine receptors, cytokines and cytokine receptors by quantitative RT-PCR at baseline and week 12 after r-hIL-7 administration. Increases in mRNA levels were only noted for the following chemokine and cytokine related genes: *CCRL1* (chemokine receptor like 1), *CMTM1* (CKLF-like MARVEL transmembrane domain containing 1), *TNFSF14* (TNFSF14 Tumor necrosis factor ligand superfamily member 14) and *IL-17B* (data not shown).

### Effect of r-hIL-7 on viral persistence in the gut

Since increases in T-cells in the gut were observed, DNA was extracted from gut tissue for proviral DNA measurements. There were no statistically significant changes in the number of copies of proviral DNA (median of 16 copies/10^6^ cells at baseline vs 28 copies/10^6^ cells at week 12, P = 0.078, supplemental Figure S7). Proviral DNA level was below detection in one subject at both tested time points.

### Decreased plasma levels of D-dimer and sCD14, and phenotypic changes of peripheral blood monocytes after r-hIL-7

**Figure 6:**
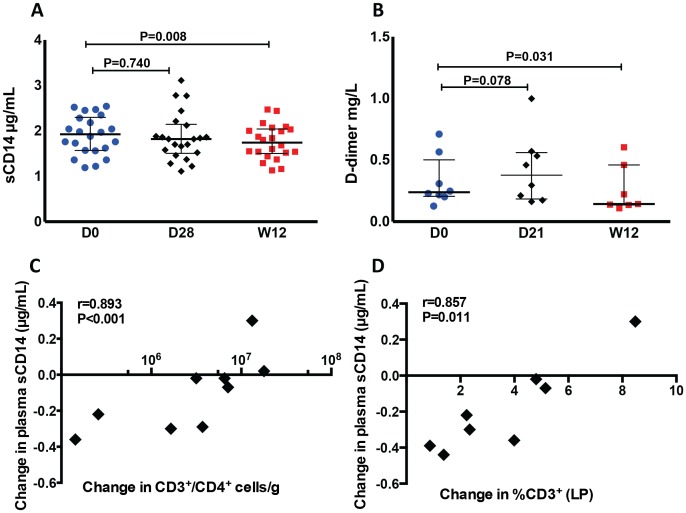
Decreased plasma levels of sCD14 and D-dimer after r-hIL-7 administration. Plasma levels of sCD14 (A) and D-dimer (B) decreased significantly at week 12 after r-hIL-7 compared to baseline (D0) after a transient and not statistically significant increase at day 21 or 28 after the first injection. Day 0 was the day of the first r-hIL-7 injection. Significant associations were seen between the changes in plasma sCD14 and the changes in CD4^+^ T-cells in colonic mucosa as measured by flow cytometry (C) and with the CD3^+^ changes observed by IHC (D).

As shown in Figure 6A, plasma levels of sCD14 decreased from a baseline value of 1.925 mg/L to 1.740 mg/L at week 12 (P = 0.010, Figure 6A). Levels of D-dimer also decreased from a baseline value of 0.238 mg/L to 0.143 mg/L at week 12 (P = 0.031) after a transient, not statistically significant, increase to 0.377 mg/L at day 21 (P = 0.741) of r-hIL-7 administration. Changes in plasma sCD14 strongly correlated with changes of CD4^+^ T-cells in the gut mucosa as measured by flow cytometry (r = 0.893, P<0.001, Figure 6C) as well as with the CD3^+^ surface area staining changes in LP as measured by IHC (r = 0.857, P = 0.011, Figure 6D).

Because sCD14 is a marker of monocyte activation, we also assessed the expression of markers of migration (CCR2, CX3CR1) and activation (CD16) on monocytes at day 0 (start of study drug), day 28 and week 12 of r-hIL-7 administration. A small decrease of CCR2 expression (which co-localizes with CD14) was observed at week 12 (from a median of 89.80% of monocytes at baseline to 88.00%, P = 0.014), concomitantly with an increase in CX3CR1 expression (from a median of 5.07% of monocytes at baseline to 6.70% at week 12, P = 0.043, Figure S8A and S8B). A small decrease in the proportion of intermediate (CD14^+^CD16^+^) monocytes was noted at week 12 compared to baseline (1.06 from 1.39%, P = 0.074, Figure S8C). Expression of CD127 on monocytes did not change significantly at any of the tested time points. Compared to baseline, monocytes were found to have a lower basal (in the absence of stimulation) IL-1β expression at week 12 (4.56% vs 6.11% of monocytes, P = 0.018, Figure S8D) while TNF expression remained unchanged (P = 0.900). Monocyte production of these cytokines after LPS stimulation was not different at week 12 compared to baseline.

## Discussion

In this study, the effects of in vivo administration of r-hIL-7 on the colonic mucosa were evaluated. Peripheral blood T-cell expansions were observed as previously reported [21], including an expansion of T-cells expressing the gut homing integrin α4β7. Rectosigmoid biopsies showed that peripheral T-cell expansions were accompanied by T-cell increases in the colonic mucosa. Importantly, we also observed a decrease in lamina propria neutrophil infiltration and TNF staining as well as a decrease in plasma levels of sCD14 and D-dimer, biomarkers that have been linked to morbidity and mortality in HIV infection. These findings suggest a potential role for r-hIL-7 in restoring mucosal integrity and decreasing local and systemic inflammation in patients with HIV infection on ART.

Peripheral T-cell expansions were seen in earlier studies of r-hIL-7 in humans with HIV or cancer [22], [23], [29]. Mucosal CD4^+^ T-cells are severely depleted in HIV infection and are not always fully restored in the lamina propria with ART [30]. We had previously shown that IL-2 administration did not affect gut mucosal T-cell numbers despite significant expansions in the peripheral blood that did not confer clinical benefit. In vitro and in vivo studies in mice and macaques have shown that IL-7 may increase homing of T-cells to the gut and local cycling [24], [25]. Testing this hypothesis in humans, we indeed confirmed an increase of T-cells in the gut by both flow cytometry and IHC. Interestingly, the increases of mucosal CD4^+^ T-cells correlated strongly with the expansion of CD4^+^α4β7^+^ T-cells in peripheral blood while this was not observed for mucosal CD8^+^ T-cells that were strongly associated with local CD8 cycling. These data support recent observations that trafficking and T cell dynamics in the gut mucosa may differ significantly between CD4^+^ and CD8^+^ T-cells [12] and could also represent functional differences that were not addressed in our study.

A subset of participants who underwent rectosigmoid biopsies had tissue evaluated by both flow cytometry and IHC with strong correlation of CD3^+^ measurements between the two methods. Flow cytometry showed more dramatic CD4^+^ T-cell increases in the mucosa and relative normalization of CD4^+^ T-cell numbers, while IHC showed increases of CD8^+^ cells in the LP suggesting that the expansion or trafficking of CD4^+^ T-cells may have been more prominent in the lymphoid follicles (inductive sites) than within the LP. This may not be surprising as the mucosal CD4^+^ T-cell expansion after IL-7 administration was more pronounced among naïve subsets driven by enhanced homing while CD8^+^ T-cell expansion may have been driven by local cycling. Another possibility is that the threshold of CD4 intensity used to identify CD4^+^ T-cells by IHC may have been less sensitive compared to flow cytometry using both CD3 and CD4 antibodies. Our study was limited by the fact that the material available for IHC was not sufficient to allow evaluation of lymphoid follicles to confirm this hypothesis.

Regardless, neutrophil infiltration in the lamina propria, which was higher in patients than among controls at baseline, decreased significantly after r-hIL-7, albeit not to normal levels. Similar dramatic decreases after r-hIL7 administration were found in TNF surface staining in the lamina propria, which was significantly higher in HIV+ patients compared to controls. In contrast, intracellular FOXP3 surface staining was much lower in HIV+ participants than among controls and increased after IL-7 treatment probably as a consequence of a higher number of mucosal CD4^+^ T-cells. It is known from animal models of colitis that regulatory CD4^+^ T-cells can both prevent and ameliorate inflammatory colitis [31]. In addition, in ulcerative colitis in humans, lamina propria TNF, originating from T-cells and macrophages, strongly correlates with grade of inflammation [32]. Although the source of TNF in our study was unclear and could represent T-cells, monocytes, macrophages or neutrophils, our findings point to a significant repair of the mucosal injury and decrease in the local inflammation that persists in chronic ART-treated HIV infection.

Exploratory evaluation revealed increases in mRNA of certain chemokines, chemokine receptors and cytokines (IL-17) that favor both gut trafficking of T-cells and improved epithelial integrity [13] which could be a direct effect of IL-7 or a secondary effect of the increased T-cells in the colonic mucosa. CCRL1 (chemokine receptor like 1) can bind CCL19, CCL20 and CCL25 and may have a role in epithelial integrity, homing to gut tissue and response to enteric bacterial pathogens [33]–[35]. Similarly, TNFSF14 or LIGHT has critical roles in lymph node homing, inflammation and wound healing [36], [37]. Our study had limited availability of tissue for additional experiments studying the role of these pathways but these preliminary findings suggest that r-hIL-7 may have affected trafficking of T cells in the colonic mucosa and may have promoted local tissue repair. Timing of biopsies closer to administration would have been more revealing with respect to induction of chemokine and chemokine receptor alterations as shown in animal studies [24], [25] but could have significantly increased inconvenience and hindered study participation.

The design of this study was not to address possible implications of r-hIL-7 in tissue or cell reservoirs, which is a complex issue. Earlier studies have suggested a role for IL-7 in maintaining a reservoir of latently integrated virus within central memory CD4^+^ T-cells [38]. Since there was a significant increase of CD4^+^ T-cells in the gut mucosa (presumably mostly in lymphoid follicles), total proviral gut DNA was evaluated in gut tissue specimens of study participants at baseline and week 12 after r-hIL7. There were no significant changes noted and importantly, one participant without detectable proviral DNA at baseline had no detection of proviral DNA after r-hIL7. These results would suggest a lack of dramatic perturbation in the gut reservoir although studies focusing on this specific question could be more revealing.

Despite the significant correlation of indices of microbial translocation with T-cell activation and immune restoration, levels of microbial products in circulation have not been linked to clinical events. In contrast, markers of monocyte activation, typically in response to LPS (sCD14), and coagulation markers (D-dimer) have been found to be strong independent predictors of clinical events in patients treated successfully with ART [8], [9], [39]. Evaluation of these biomarkers in this study showed a small yet statistically significant decline at week 12. The changes in plasma sCD14 were strongly associated with the changes in T-cells in mucosa by both used methods, establishing a tight link between alterations in mucosal T-cells and systemic inflammation. Based on these findings and recent recognition of the role of monocytes in chronic inflammation with HIV [40], we also found decreases in inflammatory monocytes and a decrease in their basal (unstimulated) IL-1β expression, one of the main proinflammatory cytokines produced by monocytes. Interestingly, both IL-1β and TNF play a prominent role in extra-intestinal thrombosis in animal models of colitis [41], [42]. These findings highlight r-hIL-7 effects beyond T-cells that warrant further investigation in future studies.

In summary, administration of r-hIL-7 expands peripheral T-cells expressing α4β7 and leads to increased colonic mucosal T-cells. This increase of T-cells in the mucosa is associated with a significant increase in FOXP3^+^ T-cells and a decrease in gut neutrophil infiltration and local TNF production, and is strongly linked to decreases in levels of inflammatory and coagulation biomarkers. The clinical implications of these observations remain uncertain but strongly suggest a potential role for r-hIL-7 in repairing the mucosal injury and innate system activation that persist in chronic treated HIV infection.

## Methods

### Clinical trial design

INSPIRE2 was a single arm clinical trial conducted in US (Case Western Reserve, NIH/intramural NIAID, University of Miami) and Canada (McGill University Health Centre). The study was approved by the ethics committees of the participating institutions (University Hospitals Case Medical Center, NIAID, University of Miami and McGill health Centre), and all subjects signed informed consent at screening. The study was registered in clinicaltrials.gov, NCT01190111. Eligible participants had to be receiving ART for a minimum of one year with plasma HIV-RNA<50 copies/mL and with a CD4^+^ T-cell count between 101–400 cells/µL. Patients with chronic hepatitis B or C or seropositive for HIV-2 or HTLV 1 or 2 were excluded. A sample size of 23 was selected to allow adequate safety information and conduct immunologic studies. The dose given was based on the previous dose finding clinical trial of a similar formulation of r-hIL-7 (study CLI-107-06, INSPIRE 1) and consisted of a cycle of 3 subcutaneous injections at 20 µg/kg administered once every week for 3 consecutive weeks [26].

### Immunophenotyping of peripheral blood mononuclear cells (PBMC)

Flow cytometric analyses were performed on cryopreserved PBMCs. For peripheral blood T-cell staining, labeled cells were acquired on a CyanADP cytometer and analyzed using Summit software. T-cell subsets were defined using the following antibodies: Live/dead-PO, CD3-PB (Clone:SP34-2), CD4− PerCP-Cy5.5 (Clone:L200), CD8-APC, CD45RA-PE or CD45RA-APC (Clone:HI100), CCR7-PE-Cy7 (Clone:3D12), Integrin β7 FITC R&D (Clone:FAB4669F), CD49d (α4) PE (Clone:L25 BD), FOXP3-Alexa488 (Clone:259D/C7) and CD25-APC (Clone:2A3) from BD Biosciences. Immunophenotyping assessments were performed at D0, D28 and week 12. Monocyte phenotyping, stimulation with lipopolysaccharide (LPS), and cytokine quantification were also performed on cryopreserved PBMCs. Cells were thawed and stimulated for 6 h with 1 µg/mL lipopolysaccharide (LPS) (Invitrogen, Carlsbad, CA) in the presence of 1 µL/mL of brefeldin A (Sigma-Aldrich) to prevent cytokine release. Cells were washed in RPMI media containing 10% heat-inactivated human serum and then stained with a Live/Dead fixable blue dead cell stain (Invitrogen). The cells were then washed in phosphate buffered saline (PBS) and stained with the following antibodies: anti-CD2 efluor450 (Clone:RPA-2.10), anti-CD3 efluor450 (Clone:UCHT1), anti-CD19 efluor450 (Clone:HIB19), anti-CD20 APC (Clone:2H7), anti-IL-1β PE (Clone:CRM56), and anti-HLADR efluor605 (Clone:LN3) from eBioscience; anti-CD16 PE-Cy7 (Clone:3G8), anti-CX3CR1 APC (Clone:2A9-1), anti-CCR2 PerCP-Cy5.5 (Clone:TG5) and anti-TNF PerCP-Cy5.5 (Clone:MAB11) from BioLegend; anti-CD14 PE (Clone:M5E2), anti-CD56 PacBlue (Clone:B159), anti-HLA-DR APC-Cy7 (Clone:R22.32), and anti-CCR5 APC-Cy7 (Clone:2D7) from BD Biosciences; and anti-CD127 PE (Clone:R34.34) from Beckman Coulter. Cells were washed in PBS with 1% BSA and fixed according to the eBioscience Fix/Perm protocol (eBioscience). Labeled cells were assessed with an LSRII cytometer and analyzed using FlowJo software, version 9.5.2 (Tree Star). Only live cells were included in all analyses; samples with viability <75% were not included.

### Gut biopsy specimen processing

A subgroup of study participants at McGill University Health Centre and intramural NIAID underwent rectosigmoid biopsies. All gut biopsy participants were clinically asymptomatic at the time of the procedures. Contemporaneous HIV^−^ controls (N = 10) were recruited at the same two sites, HIV^−^ volunteers had a median age of 53 years and 6 of 10 were male.

Biopsies were performed between 1 to 3 weeks prior to any drug administration (referred as “baseline” in figures) and at week 12. All biopsy specimens were processed in the same laboratory at intramural NIAID. Gut (rectosigmoid) random biopsies were performed during routine flexible sigmoidoscopy and processed as previously described [11], [43]. Briefly, approximately twenty-five to thirty biopsies were randomly extracted with 16–20 processed for flow cytometric analysis. In groups of five, the biopsies were weighed, placed in 500 µL of medium containing RPMI (Mediatech, Herndon, VA) with 10% human serum (Gemini BioProduct). Samples were then digested using 0.5 mg collagenase (Sigma-Aldrich) and 250 U benzonase (Novagen) for 40 min at 37°C before being filtered through a 40 micron screen. After being washed twice with the 10% human serum medium, the resulting cell suspension was counted using a Beckman Coulter Counter to obtain the number of total viable cells. Absolute numbers of CD4^+^ and CD8^+^ T-cells per gram of gut tissue were calculated by dividing the viable cell count by the tissue weight. This number was then multiplied by percentages obtained from flow cytometric analysis (see below) to determine the absolute cell count of the T-cell subsets.

### Gut biopsy specimen flow cytometric methods

Cryopreserved PBMC and cells extracted from the gut biopsy specimens were rested overnight at 37°C before stimulation for 6 h with 40 ng/ml of phorbol myristate acetate (PMA) and 1 µM ionomycin in the presence of 1 µL/mL of brefeldin A (Sigma-Aldrich) to prevent cytokine release. After two washes with RPMI medium containing 10% heat-inactivated human serum (Gemini BioProduct), cells were fixed and permeabilized according to the eBioscience Fix/Perm protocol. The cells were stained with the following antibodies: anti-CD3 PE-Cy7 (Clone:SK7), anti-CD4 APC-Cy7 (Clone:SK3), anti-CD8 PacBlue (Clone:RPA-T8), anti-CD8 PerCP (Clone:SK1), anti-CD27 FITC (Clone:M-T271), anti-CD45RO APC (Clone:), anti-tumor necrosis factor (TNF) APC (Clone: 6401.1111), anti-Ki67 FITC (Clone:MOPC-21) from BD; anti-interferon γ (IFN-γ) PacBlue (Clone:4S.B3) and anti-IL-17 PE (Clone:ebio64Dec17) from eBioscience. Samples were acquired (approximately 200,000 events per sample) and analyzed as described above. In the gut mucosa, T-cells that were CD45RO^+^/CD27^+^ were identified as central memory (CM) and CD45RO^+^/CD27^−^ as effector memory (EM).

### Immunohistochemistry and quantitative image analysis

Immunohistochemistry (IHC) was performed in a subset of eight study participants who had sufficient tissue (two to three pieces from rectosigmoid biopsies) available for tissue fixation. A biotin-free polymer approach (Golden Bridge International, Inc.) was used on 5-µm tissue sections mounted on glass slides, which were dewaxed and rehydrated with double-distilled H2O. Heat induced epitope retrieval (HIER) was performed by heating sections in 0.01% citraconic anhydride containing 0.05% Tween-20 or 0.01M Tris-HCL pH 8.6 in a pressure cooker set at 122–125°C for 30 s. Slides were incubated with blocking buffer (TBS with 0.05% Tween-20 and 0.25% casein) for 10 min. For CD4 and CD8 IHC, slides were incubated with mouse anti-CD68 (1∶400; clone KP1, Dako), mouse anti-CD163 (1∶400; clone 10D6; Novocastra/Leica) and rabbit monoclonal anti-CD4 (1∶200; clone EPR6855; Epitomics, Inc.) or mouse anti-CD4 (1∶100; clone 1F6; Vector Laboratories) and rabbit monoclonal anti-CD8 (1∶50; clone SP16; Labvision/Thermo Scientific) diluted in blocking buffer over night at 4°C. Slides were washed in 1× TBS with 0.05% Tween-20 and endogenous peroxidases blocked using 1.5% (v/v) H2O2 in TBS (pH 7.4) for 10 min. Slides were incubated with Mouse Polink-1 AP followed by Rabbit Polink-1 HRP or Mouse Polink-1 HRP followed by Rabbit Polink-1 AP for 30 min at room temperature. Sections were first incubated with Impact™ DAB (3,3′-diaminobenzidine; Vector Laboratories) to develop the CD4, washed and developed with Warp Red (Biocare Medical, Inc.) to either visualize the CD8^+^ T-cells or mask the faint CD4 expressed on APCs to distinctly identify CD4^+^ T-cells. For CD3, myeloperoxidase (MPO), TNF and FOXP3 IHC, after the HIER step slides were loaded on an IntelliPATH autostainer (Biocare Medical) and stained with optimal conditions determined empirically that consisted of a blocking step using blocking buffer (TBS with 0.05% Tween-20 and 0.25% casein) for 10 minutes and an endogenous peroxidase block using 1.5% (v/v) H_2_O_2_ in TBS (pH 7.4) for 10 minutes. Rabbit monoclonal anti-CD3 (1∶100; clone SP7; Labvision/Thermo Scientific), rabbit polyclonal anti-myeloperoxidase (1∶1,000; Dako), mouse monoclonal anti-TNF antibody (1∶1,000; clone P/T2; Abcam) and mouse monoclonal anti-FOXP3 antibody (1∶100; clone 236A/E7; Abcam) were diluted in blocking buffer and incubated for 1 h at room temperature. Tissue sections were washed, and detected using the Rabbit Polink-1 HRP (CD3 and MPO), Mouse Polink-1 HRP (TNFα) or Mouse Polink-2 HRP (FOXP3) staining systems (Golden Bridge International, Inc) according to manufacturer's recommendations. Sections were developed with Impact DAB (Vector Laboratories). All slides were washed in ddH2O, counterstained with hematoxylin, mounted in Permount (Fisher Scientific), and scanned at high magnification (×200) using the ScanScope CS System (Aperio Technologies) yielding high-resolution data from the entire tissue section. Representative regions of interest (ROIs; 500 µm^2^) were identified and high-resolution images extracted from these whole-tissue scans. The percent area of the lamina propria that stained for CD3^+^ cells, CD4^+^ cells (excluding non T-cells), CD8^+^ cells, myeloperoxidase^+^, TNF^+^ and FOXP3^+^ cells were quantified using Photoshop CS5 and Fovea tools.

### Measurement of total proviral DNA in gut biopsy specimens and PCR array analysis

DNA from gut biopsy samples was isolated using the DNA isolation kit from Epicenter. HIV DNA level was estimated by real-time PCR as previously described [44]. HIV copies were normalized to the level of RNAseP gene copies estimated by real-time PCR.

OCT-embedded gut tissue samples were removed from frozen blocks using a small amount of RNA later (Life Technologies). The biopsy samples were homogenized in RLT buffer (RNeasy Mini Kit, QIAGEN) using a handheld rotor-stator homogenizer. Total RNA was extracted by an RNeasy Mini Kit (QIAGEN) and treated with DNase Ι to eliminate possible genomic DNA contamination. RNA quantity and purity were determined using the NanoDrop ND-2000 spectrophotometer (Nanodrop Technologies), and RNA integrity was rated according the RNA integrity number (RIN) using the Bioanalyzer 2100 capillary electrophoresis system (Agilent Technologies). The average RIN for the OCT-embedded gut tissue samples was 7.5 (range 6.7–8.3).

The expression profile of common cytokine-related genes (84 genes) and chemokine-related and its receptor-related genes (84 genes) was determined using a 96-well format Human Common Cytokine RT2 Profiler PCR array (PAHS-021) and Human Chemokines and Receptors RT2 Profiler PCR array (PAHS-022) according to the manufacturer's instructions (SABiosciences/QIAGEN). The array also included five housekeeping genes (HKG). qPCR was run on the 7900HT Fast Real-Time PCR System (Applied Biosystems/Life technologies) equipped with SDS 2.3 software, using RT2 SYBR Green/ROX qPCR master mix (SABiosciences/QIAGEN). Data analysis was done by the ΔΔCt method using the MS-Excel sheet with macros downloaded from the manufacturer's website (SABiosciences/QIAGEN). A small group of genes that were differentially expressed between the pre- and post-IL-7 therapy time points (P value<0.05 and fold change >2 based on parametric testing, paired t-test) were identified and used for plots (Supplemental Figure S4).

### Plasma biomarker measurements

Cryopreserved plasma was processed for sCD14 measurements in duplicate using a commercially available ELISA assay (R&D Systems, Minneapolis, MN), and analyzed according to manufacturer's recommended procedure. D-dimer levels were quantified with an enzyme linked fluorescent assay (BioMérieux Inc., Durham, North Carolina, USA).

### Statistical methods

Values presented are medians with interquartile ranges and non-parametric testing was used for comparisons throughout. Mann-Whitney tests were used to compare the HIV+ group (either at baseline or at timepoints after r-hIL-7 therapy) with the HIV− controls. Paired Wilcoxon signed rank was used to compare matched paired values before and after r-hIL-7 therapy. Spearman correlations were performed to look for associations between variables of interest. Due to the exploratory nature of the work, p-values of ≤0.05 are shown. Analyses were performed using Prism v6 (GraphPad Software, Inc., La Jolla, CA).

## Supporting Information

Figure S1 T-cell expansions in peripheral blood in both naïve and memory subsets after one r-hIL-7 cycle. Naïve (A and B) as well as central memory (C and D) and effector memory (E and F) CD4**^+^** and CD8**^+^** T-cells in peripheral blood increased significantly at week 12 after administration of r-hIL-7 (all P values<0.001) compared to D0. Day 0 was the day of the first r-hIL-7 injection.
(TIFF)

Figure S2 The fold increase of α4β7 T cells was more pronounced in naïve CD4^+^ (A) and CD8^+^ (B) T-cell subsets. The change in CD4^+^ T-cell numbers in colonic mucosa between baseline and week 12 correlated strongly (r = 0.65, P = 0.026) with the concomitant changes in CD4^+^α4β7^+^ T-cells in peripheral blood (C) but a similar association was not observed for CD8^+^ T-cells (D).
(TIFF)

Figure S3 The numbers of cycling CD4^+^ (A) and CD8^+^ (B) T-cells (expressing Ki67) in colonic mucosa increased significantly at week 12 after r-hIL-7 administration (P = 0.024 and P = 0.037 respectively, by Wilcoxon paired signed rank test). The number of mucosal CD4^+^ T-cells at week 12 did not correlate with the local (mucosal) cycling of CD4^+^ T-cells (C). In contrast, the number of mucosal CD8^+^ T-cells at week 12 correlated strongly (r = −0.90, P<0.001) with the local (mucosal) cycling of CD8^+^ T-cells (D).
(TIFF)

Figure S4 The proportion of CD4^+^ T–cells in the gut expressing IFNγ (A), TNF (B) and IL-17 (C) after stimulation with PMA/ionomycin and expressing FOXP3 (D) as described in methods. Cells were extracted from rectosigmoid biopsies performed prior to r-hIL-7 administration (baseline) and at week 12 of study.
(TIFF)

Figure S5 The percent area of the LP staining for CD4^+^ (A) and CD8^+^ (B) cells was evaluated in the LP at baseline, and at week 12, after r-hIL-7 and showed a significant increase in CD8^+^ cells but not CD4^+^ cells at week 12 compared to baseline (P = 0.008).
(TIFF)

Figure S6 The baseline/week 12 paired data for the LP staining for MPO (A), TNF (B) and FOXP3 (C). P values by Wilcoxon matched paired comparisons. A strong correlation (r = 0.89, P = 0.033) was observed between the drop of TNF in LP and the simultaneous increase of FOXP3^+^ cells/mm^2^ (D).
(TIFF)

Figure S7 Total proviral DNA was measured in gut tissue at study baseline and at week 12 after r-hIL-7. One study participant had values below the limit of detection at both time points. Median values showed no statistically significant changes at week 12 (P = 0.078).
(TIFF)

Figure S8 Monocyte phenotype and intracellular cytokine staining were performed in PBMC from day 0 (D0, first r-hIL-7 injection) and at week 12 (W12). (A) Expression of CCR2 decreased at week 12 compared to D0 (P = 0.014) with a reciprocal increase of (B) CX3CR1 expression (P = 0.043). There was a decrease in basal IL-1β expression (C) by monocytes at week 12 (P = 0.018), in the presence of unchanged TNF expression (P = 0.900) (D).
(TIFF)
