# Supplementary figures and images for: Decreases in Colonic and Systemic Inflammation in Chronic HIV Infection after IL-7 Administration
Source: PLoS Pathog. 2014 Jan 30;10(1):e1003890. doi: 10.1371/journal.ppat.1003890 (PMC3907377; doi:10.1371/journal.ppat.1003890)

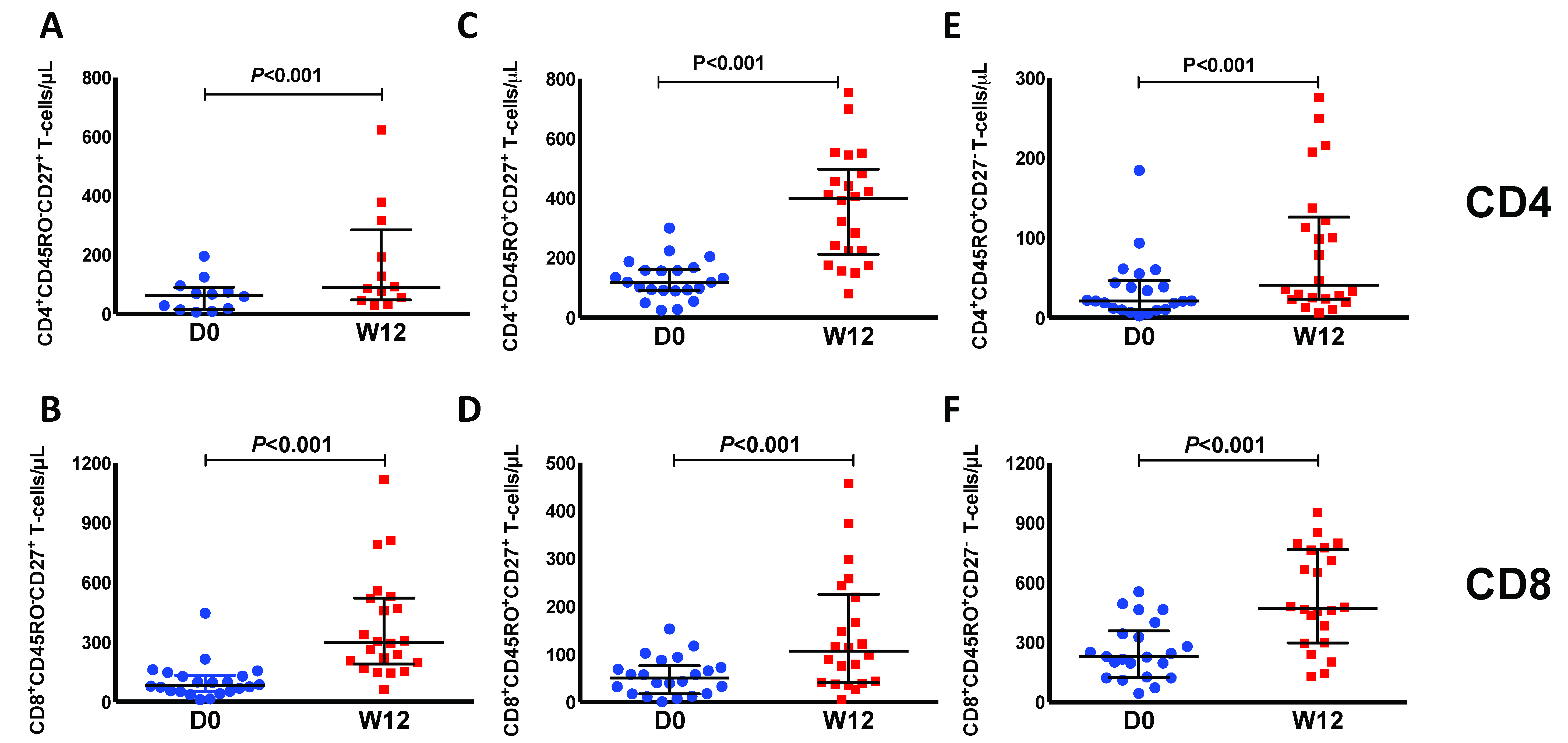

Supplement: Figure S1 — T-cell expansions in peripheral blood in both naïve and memory subsets after one r-hIL-7 cycle. Naïve (A and B) as well as central memory (C and D) and effector memory (E and F) CD4+ and CD8+ T-cells in peripheral blood increased significantly at week 12 after administration of r-hIL-7 (all P values<0.001) compared to D0. Day 0 was the day of the first r-hIL-7 injection. (TIFF) [file ppat.1003890.s001.tiff]

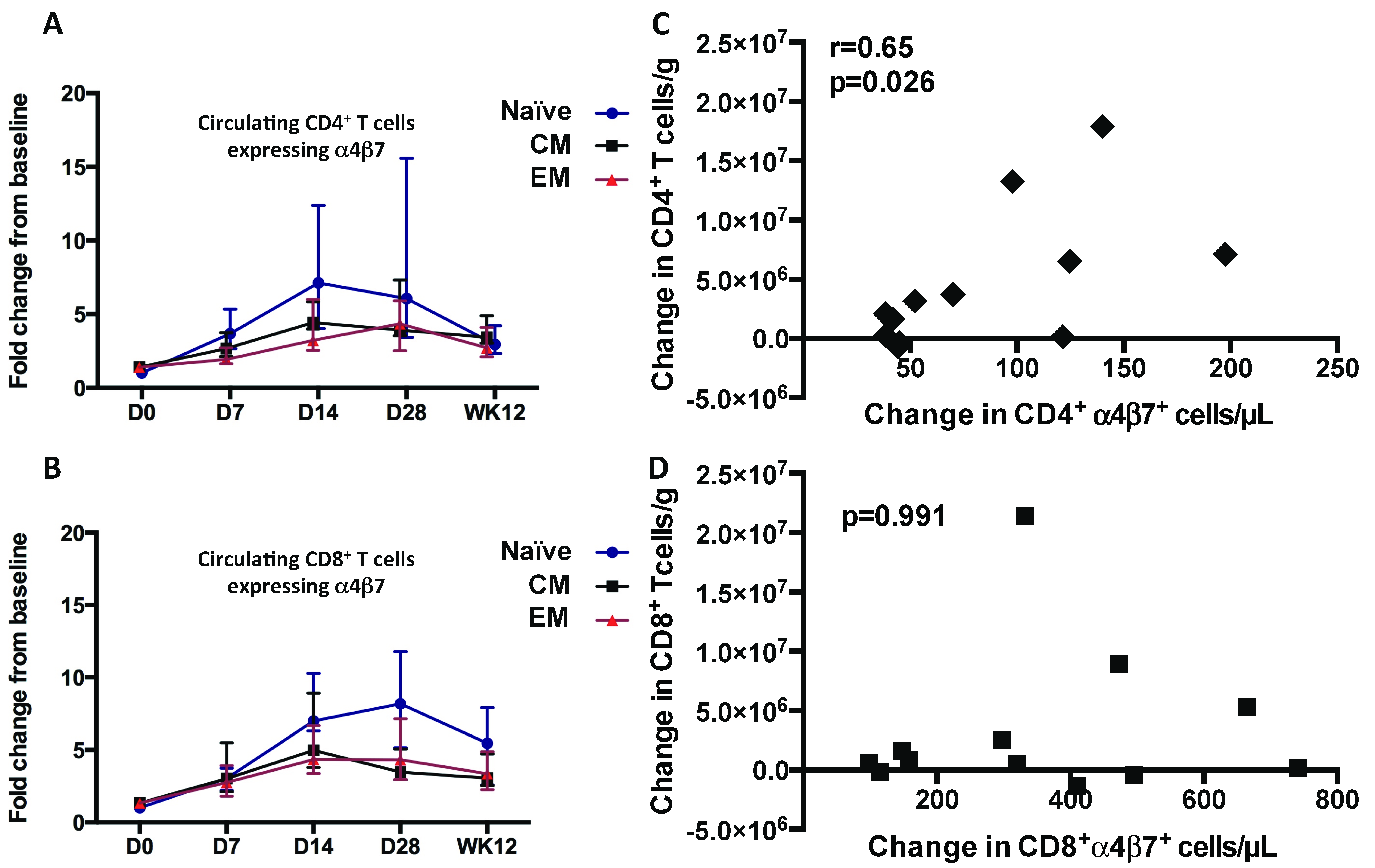

Supplement: Figure S2 — The fold increase of α4β7 T cells was more pronounced in naïve CD4+ (A) and CD8+ (B) T-cell subsets. The change in CD4+ T-cell numbers in colonic mucosa between baseline and week 12 correlated strongly (r = 0.65, P = 0.026) with the concomitant changes in CD4+α4β7+ T-cells in peripheral blood (C) but a similar association was not observed for CD8+ T-cells (D). (TIFF) [file ppat.1003890.s002.tiff]

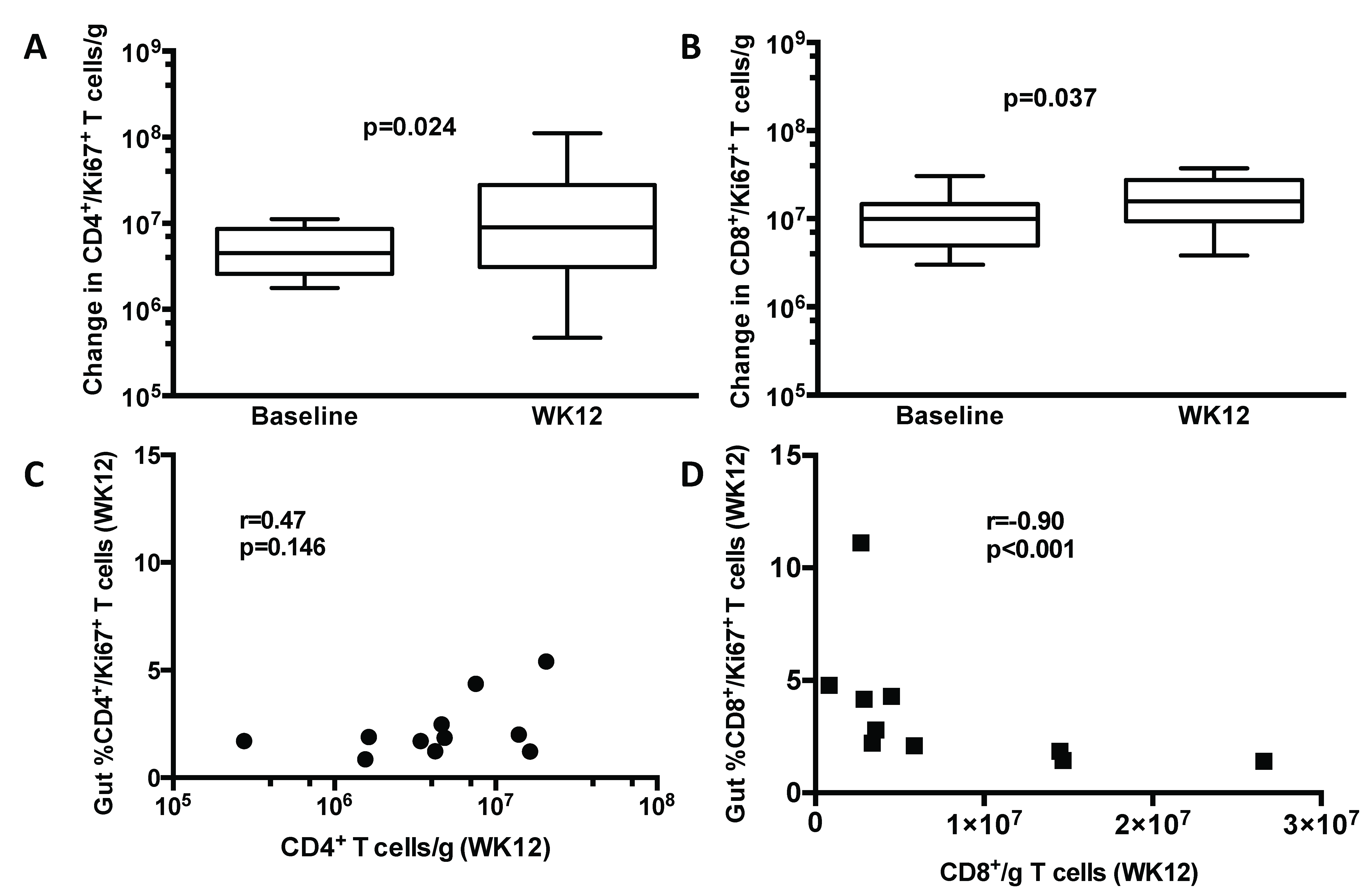

Supplement: Figure S3 — The numbers of cycling CD4+ (A) and CD8+ (B) T-cells (expressing Ki67) in colonic mucosa increased significantly at week 12 after r-hIL-7 administration (P = 0.024 and P = 0.037 respectively, by Wilcoxon paired signed rank test). The number of mucosal CD4+ T-cells at week 12 did not correlate with the local (mucosal) cycling of CD4+ T-cells (C). In contrast, the number of mucosal CD8+ T-cells at week 12 correlated strongly (r = −0.90, P<0.001) with the local (mucosal) cycling of CD8+ T-cells (D). (TIFF) [file ppat.1003890.s003.tiff]

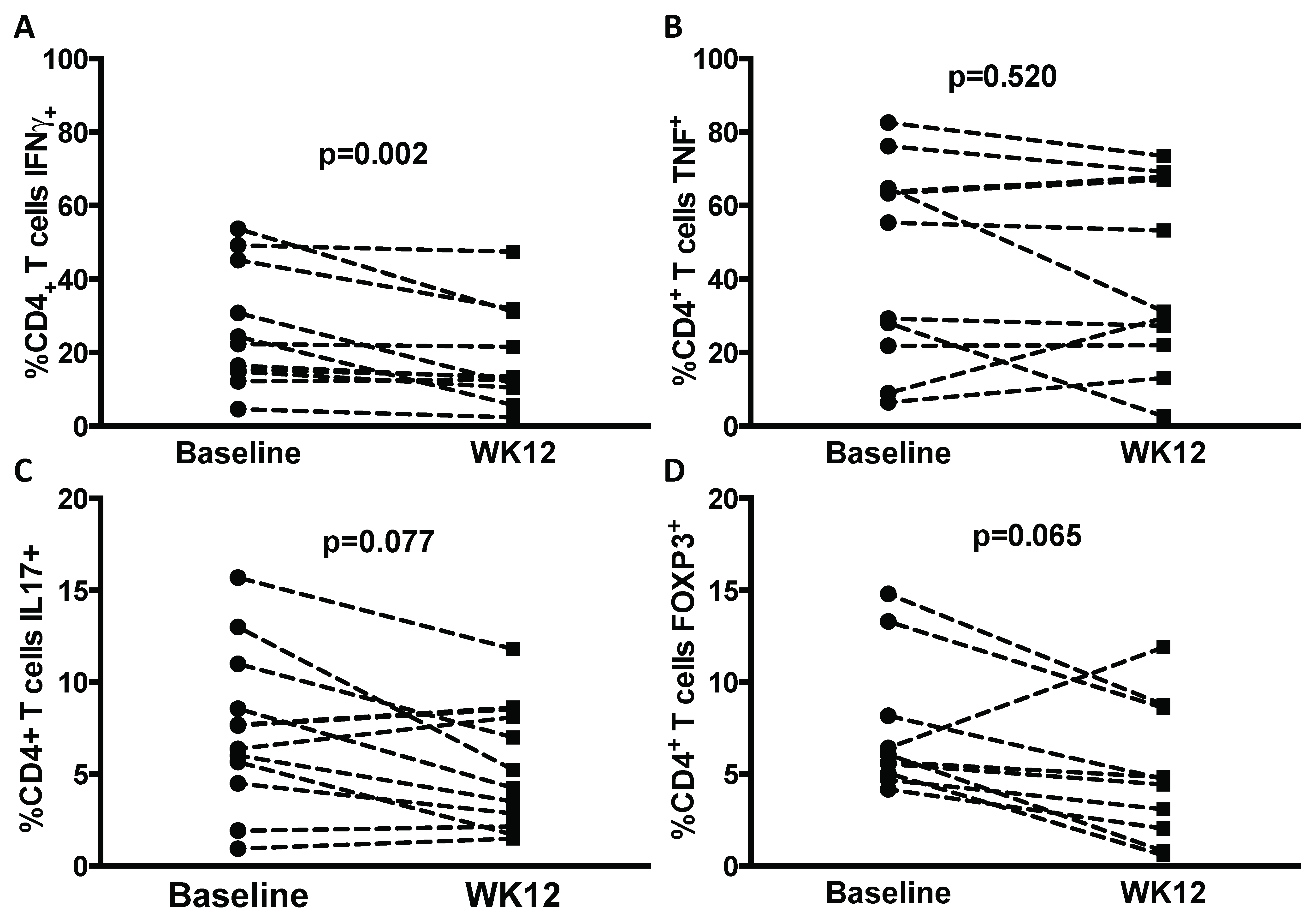

Supplement: Figure S4 — The proportion of CD4+ T–cells in the gut expressing IFNγ (A), TNF (B) and IL-17 (C) after stimulation with PMA/ionomycin and expressing FOXP3 (D) as described in methods. Cells were extracted from rectosigmoid biopsies performed prior to r-hIL-7 administration (baseline) and at week 12 of study. (TIFF) [file ppat.1003890.s004.tiff]

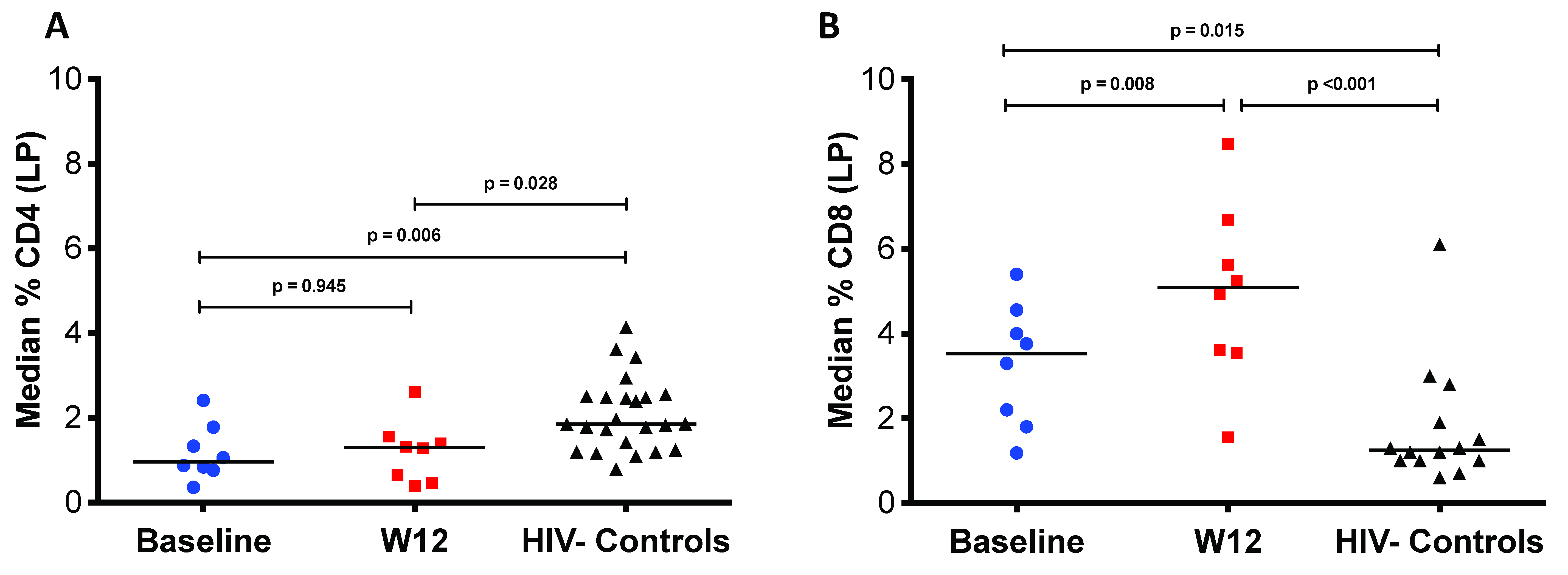

Supplement: Figure S5 — The percent area of the LP staining for CD4+ (A) and CD8+ (B) cells was evaluated in the LP at baseline, and at week 12, after r-hIL-7 and showed a significant increase in CD8+ cells but not CD4+ cells at week 12 compared to baseline (P = 0.008). (TIFF) [file ppat.1003890.s005.tiff]

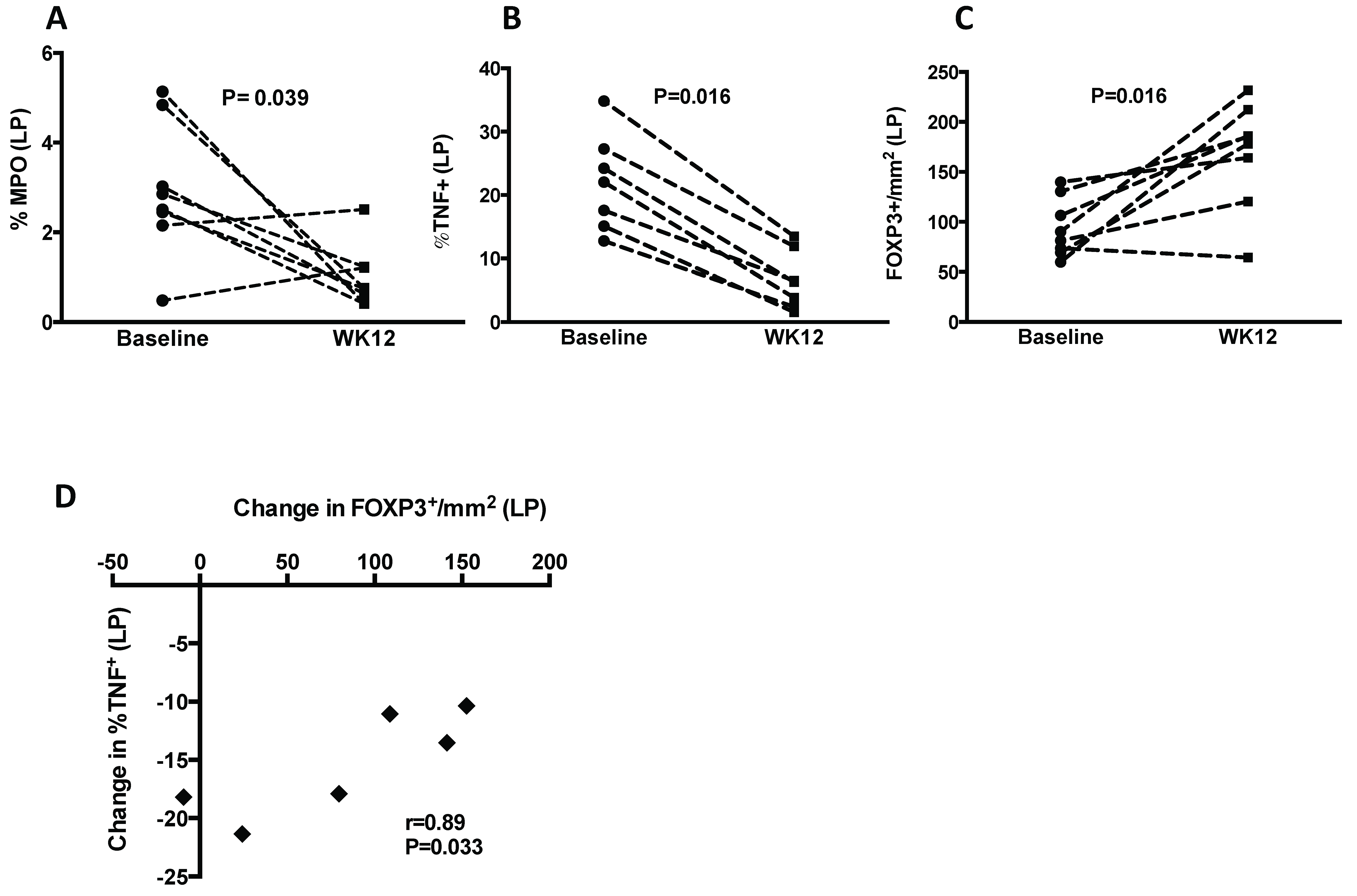

Supplement: Figure S6 — The baseline/week 12 paired data for the LP staining for MPO (A), TNF (B) and FOXP3 (C). P values by Wilcoxon matched paired comparisons. A strong correlation (r = 0.89, P = 0.033) was observed between the drop of TNF in LP and the simultaneous increase of FOXP3+ cells/mm2 (D). (TIFF) [file ppat.1003890.s006.tiff]

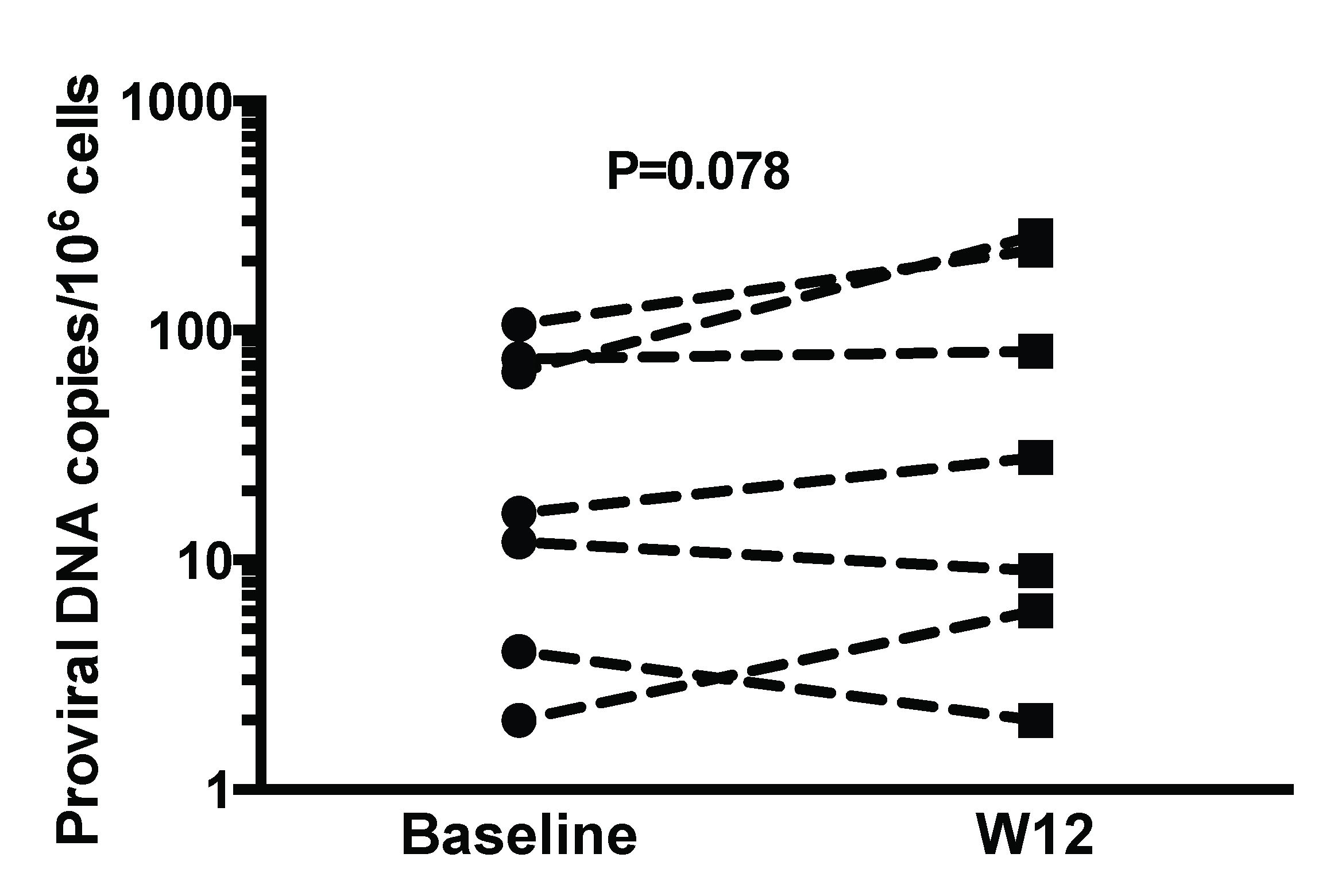

Supplement: Figure S7 — Total proviral DNA was measured in gut tissue at study baseline and at week 12 after r-hIL-7. One study participant had values below the limit of detection at both time points. Median values showed no statistically significant changes at week 12 (P = 0.078). (TIFF) [file ppat.1003890.s007.tiff]

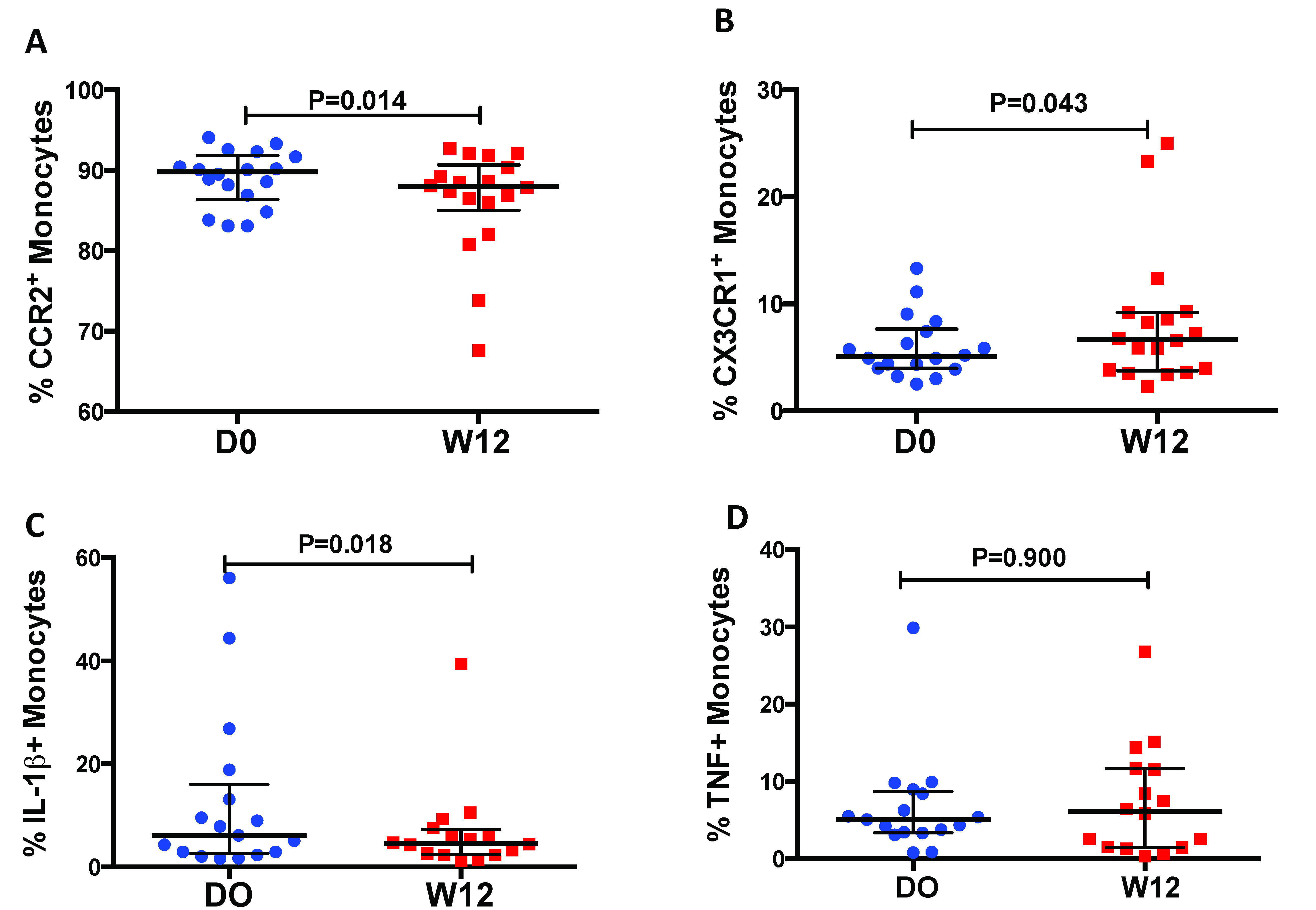

Supplement: Figure S8 — Monocyte phenotype and intracellular cytokine staining were performed in PBMC from day 0 (D0, first r-hIL-7 injection) and at week 12 (W12). (A) Expression of CCR2 decreased at week 12 compared to D0 (P = 0.014) with a reciprocal increase of (B) CX3CR1 expression (P = 0.043). There was a decrease in basal IL-1β expression (C) by monocytes at week 12 (P = 0.018), in the presence of unchanged TNF expression (P = 0.900) (D). (TIFF) [file ppat.1003890.s008.tiff]
